# Spherical Video‐Based Virtual Reality Training for Nurses’ Delirium Care Competence: A Randomized Controlled Trial With Qualitative Evaluation

**DOI:** 10.1155/jonm/8355655

**Published:** 2026-10-25

**Authors:** Yin-Ling Hung, Yi-Chao Zhou, Hsin-Pei Feng, Chia-Huei Lin, Wu-Chien Chien, Wen-Chii Tzeng

**Affiliations:** ^1^ Graduate Institute of Medical Sciences College of Medicine National Defense Medical University Taipei Taiwan medcol.mw; ^2^ Department of Nursing Tri-Service General Hospital Taipei Taiwan tsgh.ndmctsgh.edu.tw; ^3^ School of Medicine National Defense Medical University Taipei Taiwan; ^4^ Department of Family and Community Medicine Tri-Service General Hospital Taipei Taiwan tsgh.ndmctsgh.edu.tw; ^5^ Department of Health Promotion and Health Education College of Education National Taiwan Normal University Taipei Taiwan ntnu.edu.tw; ^6^ College of Nursing National Defense Medical University Taipei Taiwan; ^7^ Department of Medical Research Tri-Service General Hospital Taipei Taiwan tsgh.ndmctsgh.edu.tw

**Keywords:** competence, delirium, nursing education, simulation training, virtual reality

## Abstract

**Background:**

Delirium is an acute cognitive disturbance commonly encountered in acute care settings, and nurses play a crucial role in its early detection and management. Innovative training strategies are needed to strengthen nurses’ delirium care competence and preparedness.

**Aim:**

To determine whether a spherical video‐based virtual reality (SVVR) training program improves nurses’ knowledge, attitudes, and confidence in delirium care.

**Methods:**

A two‐group, parallel randomized controlled trial with qualitative evaluation was conducted. Registered nurses from a medical center in northern Taiwan were randomly allocated to an SVVR training group (*n* = 54) or a noninteractive video group with identical content (*n* = 54). Outcomes were assessed at baseline (T0), immediately postintervention (T1), and at 1‐month follow‐up (T2) using generalized estimating equations. Semistructured interviews with SVVR participants explored their learning experiences.

**Results:**

Both groups improved in delirium knowledge; however, the SVVR group demonstrated greater gains at T1 (*p* = 0.001) and T2 (*p* = 0.022). Confidence increased in both groups, with a larger immediate postintervention increase in the SVVR group (*p* = 0.001). Sensitivity analysis further indicated that the participants who encountered delirium cases during follow‐up reported higher confidence (*p* = 0.040). Qualitative findings showed that immersive learning enhanced engagement, concentration, perceived realism, patient assessment, clinical decision‐making, and interdisciplinary collaboration.

**Conclusion:**

SVVR training was associated with greater and sustained gains in delirium care knowledge and greater immediate gains in confidence compared with noninteractive video education. Confidence appeared to be further reinforced by subsequent clinical exposure.

**Implication for Nursing Management:**

SVVR may offer nurse managers a standardized and repeatable approach to continuing education and workforce development, particularly for preparing newly graduated nurses for delirium care. Implementation requires consideration of equipment, costs, technical support, and staff readiness.

**Trial Registration:** ClinicalTrials.gov identifier: NCT06880263

## 1. Introduction

Delirium is an acute, fluctuating neuropsychiatric condition involving disturbances in attention, awareness, and cognition [1]. It is common among hospitalized older adults, with a systematic review reporting a global prevalence of 23.6% [2]. In Taiwan, delirium was reported in approximately 75.6% of older patients receiving intensive care, which highlights its substantial burden [3]. In general, delirium is associated with increased mortality, prolonged hospitalization, complications, functional decline, and postdischarge institutional care risk [4]. Despite its multifactorial causes, early recognition and management of delirium can mitigate its consequences [5].

Nurses are well positioned to detect delirium because of their continuous patient contact. However, many nurses remain underprepared to assess and manage delirium, with limited knowledge, low confidence, unsupportive attitudes, and inconsistent use of validated assessment tools [6–8]. Although tools such as the 4AT [9], Confusion Assessment Method [10], Confusion Assessment Method for the Intensive Care Unit [11], and Intensive Care Delirium Screening Checklist [12] are available, their implementation remains inconsistent because of insufficient training, difficulty interpreting results, and perceptions that delirium screening is time‐consuming [6, 8]. In addition, delirium is unpredictable and episodic, limiting opportunities for nurses to observe, practice, and build confidence in clinical situations.

Simulation‐based learning may help address this educational gap by providing structured opportunities to practice complex clinical assessment safely [13]. Immersive approaches, including virtual reality, can enhance engagement, motivation, clinical reasoning, and learning outcomes [14]. However, conventional virtual reality platforms may be costly and technically demanding. Spherical video‐based virtual reality (SVVR), which uses 360‐degree video, offers a more accessible and affordable immersive learning approach while preserving realism and learner engagement [15]. Previous studies suggest that SVVR can improve engagement, understanding, clinical competence, skill proficiency, and knowledge acquisition in nursing and healthcare education [16–18]. However, no randomized controlled trial has evaluated SVVR training for nurses’ delirium care education.

This study was guided by Bandura’s self‐efficacy theory, which proposes that beliefs in one’s capability influence motivation, learning, and performance [19]. SVVR may support self‐efficacy through repeated practice, expert modeling, real‐time feedback, and gradual exposure to stressful clinical situations [20]. These features may strengthen nurses’ knowledge, attitudes, and confidence in delirium care beyond conventional video‐based learning. A noninteractive video with identical content was selected as the comparator to isolate the added value of immersion and interactivity [21]. Given that virtual reality may cause mild cybersickness, tolerability was also assessed [22].

Accordingly, we compared SVVR with an identical‐content noninteractive video to determine whether immersion and interactivity produced additional gains in nurses’ delirium care knowledge, attitudes, and confidence. Secondary objectives were to evaluate the tolerability and explore nurses’ learning experiences. We hypothesized that nurses receiving the SVVR intervention would demonstrate greater immediate improvements in delirium care knowledge, attitudes, and confidence than those receiving the noninteractive video intervention, and that these improvements would be sustained at 1‐month follow‐up. We further hypothesized that the SVVR training would be well tolerated and would generate positive learning experiences.

## 2. Materials and Methods

### 2.1. Settings and Design

This prospective, 1:1 parallel‐group randomized controlled trial with qualitative evaluation was conducted at a large university‐affiliated medical center in Taipei, Taiwan. The study was designed to compare an SVVR training program with a noninteractive video program containing identical delirium care content. The trial was registered at ClinicalTrials.gov on 11 March 2025 and was reported in accordance with the Consolidated Standards of Reporting Trials (CONSORT) 2025 statement. Outcomes were assessed at baseline before group assignment, immediately following the intervention, and at 1 month later. Semistructured interviews with SVVR participants were conducted to explore their learning experiences, perceived acceptability, and practical relevance of the training.

### 2.2. Participants

Participants were recruited from clinical units caring for acutely ill patients at risk of delirium, including intensive care units (ICUs), general wards, and postanesthesia recovery rooms. Participants were eligible if they were full‐time registered nurses aged 20–65 years, provided direct care, and agreed to complete all study procedures.

Nurses were excluded if they were nurse managers or held supervisory positions, did not provide direct care to patients at risk of delirium, or had conditions that could affect tolerance of virtual reality training, such as anxiety disorder, panic disorder, or claustrophobia. After providing written informed consent, the participants were randomly assigned to either the SVVR group or the noninteractive video group.

### 2.3. Intervention and Comparison

The SVVR program and noninteractive video comparator were matched in terms of clinical scenario, script, narration, learning sequence, educational content, and duration. The main differences between the two were the immersive 360° mode of presentation and the inclusion of embedded interactivity in the SVVR training. This design enabled the evaluation of the added effects of immersion and interactivity beyond those attributable to the educational content itself.

#### 2.3.1. Educational Program Development

Program development followed the Analysis, Design, Development, Implementation, and Evaluation framework [23]. Evidence‐based delirium care content was identified through literature review [5, 24, 25] and supplemented by informal interviews with five nurses regarding their educational needs, clinical challenges, and preferences for the virtual‐reality‐based training. The findings were used to design learning objectives, a clinical scenario, and a script aimed at addressing delirium assessment, safety measure analysis, risk factor identification and management, and interprofessional care.

The educational program’s content accuracy, relevance, and appropriateness were appraised by a five‐member expert panel comprising one nursing scholar, two ICU nursing supervisors, one senior psychiatrist, and one family medicine physician with expertise in medical education. The panel ratings yielded a scale‐level content validity index of 0.92. A pharmacist, a respiratory therapist, and a dietitian were consulted to refine discipline‐specific terminology and ensure the clinical accuracy of the interprofessional content.

The program comprised four modules covering delirium assessment, safety measure analysis, risk factor identification and management, and interprofessional holistic care. Each module included components of Bandura’s self‐efficacy theory, including vicarious learning, simulated mastery experiences, and verbal encouragement [19]. Embedded interactive questions were included in the SVVR version. Table 1 presents the topics, content, duration, interactive questions, and theoretical components of each module.

**TABLE 1:** Content and theoretical components of the spherical video‐based virtual reality delirium care training program.

Session	Topic	Content	Duration (min)	Interactive questions^a^	Bandura’s self‐efficacy theory
1	Delirium assessment	Delirium assessment techniques, diagnostic criteria, and symptoms related to delirium	2:19	Three questions	Mastery experiences, vicarious learning
2	Safety measure analysis	Safe protocols for delirium care	1:09	Two questions	Mastery experiences, vicarious learning
3	Risk‐factor identification and management	Underlying risk factors for delirium and appropriate delirium management strategies	2:09	Two questions	Mastery experiences, vicarious learning
4	Interprofessional care	Management approaches from interprofessional team, collaborative care planning, and communication with family members	6:58	Three questions	Mastery experiences, vicarious learning, verbal encouragement

*Note:* The training program comprised four sessions covering delirium assessment, safety measure analysis, delirium risk factors and management, and interdisciplinary holistic care. Each session included embedded interactive questions and was designed based on Bandura’s self‐efficacy theory, incorporating mastery experiences, vicarious learning, and verbal encouragement to enhance learners’ confidence in delirium care.

^a^Only for the experimental group.

Videos were captured in vacant private ICUs and conference rooms at the study hospital to protect patient privacy. All roles were played by hospital nursing staff who provided written consent to appear in the videos. A professional videographer recorded 360° footage by using an Insta360 X3 camera.

#### 2.3.2. Experimental Group: SVVR Training

Participants in the experimental group completed a single 15‐min SVVR training session on delirium care. The intervention was delivered individually in a private simulation room following a standardized protocol. The recorded footage was edited into interactive 360° modules and delivered through a head‐mounted virtual reality device (Figure 1). Participants viewed the clinical scenario through the head‐mounted display and provided real‐time spoken responses to 10 embedded interactive questions. Their responses were captured using Mandarin Chinese speech recognition technology. Fast‐forwarding and skipping were disabled to ensure the completion of the entire learning sequence. Before the beginning of the main trial, the SVVR program was pilot‐tested with 10 nurses to assess the feasibility, clarity of instructions, technical functionality, and implementation flow. These nurses were not enrolled in the randomized controlled trial.

**FIGURE 1:**
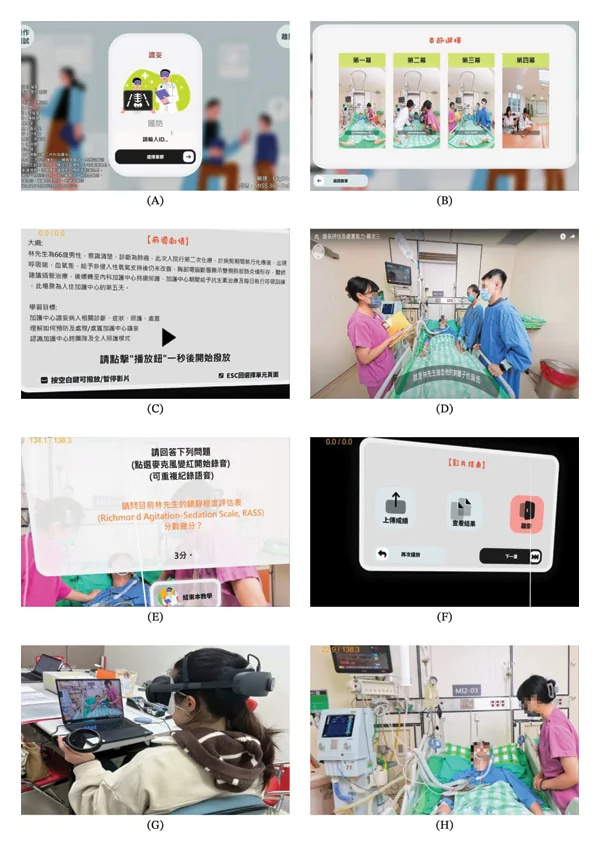
Interface, learning sequence, and delivery formats of the spherical video‐based virtual reality delirium care training program. (A) Login page; (B) chapter selection; (C) mission prologue cutscene; (D) simulated delirium care scenario in the spherical video‐based virtual reality training program; (E) interactive question; (F) performance view page, where learners could review their performance; (G) spherical video‐based virtual reality operational setup, in which learners wore a head‐mounted display to receive the training while the instructor monitored their viewing progress and interactions; and (H) comparison screenshot from the conventional two‐dimensional noninteractive video containing equivalent delirium care training content.

#### 2.3.3. Control Group: Noninteractive Video

A comparator was designed to distinguish the added effects of immersion and interactivity from those attributable to the educational content itself. Participants in the control group completed a single 15‐min session involving a conventional two‐dimensional, noninteractive video. The video had the same learning objectives, delirium care content, clinical scenario, script, narration, module sequence, and duration as those in the SVVR intervention. The participants viewed the video on a conventional computer screen in a private classroom under facilitator supervision. However, in contrast to the SVVR intervention, the video did not provide immersive 360° viewing or embedded questions requiring active participant responses. Fast‐forwarding and skipping were not permitted, and the participants were required to complete the full video.

#### 2.3.4. Usual Education

During the study period, the two groups of participants continued to receive routine hospital education; however, no additional delirium‐specific training was scheduled.

### 2.4. Outcome Measures

#### 2.4.1. Primary Outcome

Delirium care knowledge and attitudes were assessed using selected domains of the Nurse’s Assessment Ability Questionnaire in Delirium Subtypes [26]. The knowledge domain comprises 11 items, including one true‐or‐false item and 10 multiple‐choice items. Each item is scored as 1 point for a completely correct answer and 0.5 points for a partially correct answer, with a maximum score of 11. The attitude domain comprises 10 items rated on a 5‐point Likert scale, with total scores ranging from 10 to 50. Higher scores indicate greater delirium care knowledge and more positive attitudes. Face validity was evaluated to ensure linguistic appropriateness and conceptual relevance for nurses in Taiwan. In the present study, the internal consistency of the final version was satisfactory (Cronbach’s *α* = 0.85).

Confidence in assessing and managing delirium was assessed using a validated single‐item numeric rating scale: “On a scale from 0 to 10, how confident do you feel in assessing and managing patients with delirium?” [27]. Higher scores indicate greater confidence.

#### 2.4.2. Secondary Outcome

Virtual reality‐related discomfort was assessed immediately after training using the Virtual Reality Sickness Questionnaire [22]. The questionnaire comprises oculomotor (four items) and disorientation (five items) subscales, with these scored on a 4‐point Likert scale with endpoints ranging from 0 (none) to 3 (severe). Higher scores indicate greater discomfort. In the present study, internal consistency was acceptable for the oculomotor subscale (Cronbach’s *α* = 0.80), although lower for the disorientation subscale (Cronbach’s *α* = 0.53).

#### 2.4.3. Demographic and Professional Characteristics

Participants’ demographic and professional characteristics were collected at baseline, including age, gender, education level, years of nursing practice, unit type, and prior delirium care training.

#### 2.4.4. Qualitative Interviews

A semistructured interview guide adapted from Hwang et al. [28] was used to explore the participants’ learning experiences with the SVVR program after the intervention. The guide was pilot‐tested with 10 nurses to assess its feasibility, clarity, and question flow. The nurses who participated in the pilot testing were not included in the qualitative analysis. Key interview questions included (a) “How did this training differ from your previous learning experiences?”; (b) “What was the most valuable aspect of this training method? Please provide examples.”; (c) “What aspects of this training method should be improved? For example, its system functionality or interface design.”; and (d) “Would you recommend this training method to colleagues? Why or why not?” Each interview lasted approximately 15–20 min, was audio‐recorded with the participants’ permission, and was transcribed verbatim for analysis.

### 2.5. Sample Size Calculation

An a priori sample size estimation was performed using G^∗^Power version 3.1.9.7. The calculation was based on the primary outcome and was powered to detect a group‐by‐time interaction in a repeated‐measures framework with three time points. On the basis of effect sizes reported in comparable healthcare education trials [27] and the results of pilot testing, a medium effect size (Cohen’s *f* = 0.25) was specified. The calculation assumed an *α* = 0.05, power (1 − *β*) = 0.80, two groups with a 1:1 allocation ratio, a correlation of 0.50 among repeated measurements, and a nonsphericity correction of *ε* = 1.00. For a repeated‐measures analysis of variance assessing the group‐by‐time interaction, the minimum required sample size was 86 participants. To accommodate an anticipated attrition rate of approximately 25%, the recruitment target was increased to 108 participants, with 54 assigned to each group.

### 2.6. Randomization

Participants were recruited through digital advertisements on social media platforms such as Facebook, Instagram, and LINE and through institutional channels. Potential participants were asked to self‐register by using an online SurveyCake form after they were contacted by the research team through LINE to confirm their eligibility. Nurses who met the eligibility criteria and provided informed consent were enrolled.

Following completion of the baseline assessment, the participants were randomized in a 1:1 ratio to either the SVVR group or the control group. Randomization was conducted without stratification by using permuted blocks of four. The allocation sequence was generated through the Sealed Envelope web‐based randomization service (https://www.sealedenvelope.com/) by an independent research assistant who had no role in participant recruitment, intervention delivery, or outcome assessment. To maintain allocation concealment, the same assistant prepared sequentially numbered, opaque, sealed envelopes containing group assignments. Once baseline data collection had been completed, a trained clinical nurse who was not involved in recruitment or baseline assessment opened the corresponding envelope in numerical order and informed the participant and intervention team of the assigned group.

Because of the nature of the educational interventions, the participants and intervention team could not be blinded to group allocation. Outcome data were collected using self‐administered online questionnaires, and all personal identifiers were removed before data analysis.

### 2.7. Data Collection

Participant enrollment began on April 9, 2025, and data collection was completed in July 2025. The participants completed online questionnaires at baseline, immediately after the intervention, and 1 month after the intervention. These questionnaires captured their demographic characteristics and the intervention outcomes. The participants in the experimental group also completed the Virtual Reality Sickness Questionnaire immediately after training. They were then invited to participate in semistructured interviews to explore their experiences regarding the SVVR intervention.

### 2.8. Data Analysis

Quantitative data were analyzed using IBM SPSS Statistics version 25. Baseline group differences were examined using chi‐square tests or Fisher’s exact tests for categorical variables and independent *t* tests for continuous variables. The level of statistical significance was set at *p* < 0.05.

Analyses were conducted according to the intention‐to‐treat principle. Longitudinal intervention effects were estimated using generalized estimating equations (GEE) with an autoregressive first‐order correlation structure [29]. The control group and baseline assessment served as reference categories, and any covariates showing baseline imbalance were included as adjustment variables. Estimates were presented as regression coefficients (*B*) with 95% confidence intervals (*CIs*).

A sensitivity analysis was conducted to examine whether real‐world delirium care experience during the 1‐month follow‐up influenced confidence outcomes. Delirium care experience during follow‐up was entered as a binary covariate in the GEE model, and both its main effect and interaction with time were examined.

Thematic analysis followed Braun and Clarke’s six‐phase framework [30]. Two researchers independently read the transcripts and generated initial codes. They subsequently compared coding decisions, resolved differences through discussion, and iteratively refined the themes. The themes were named through discussion within the research team. Investigator triangulation and a documented audit trail were used to strengthen trustworthiness. Representative quotations were selected to support the identified themes.

### 2.9. Ethical Approval Statement

Institutional review board approval was obtained before participant enrollment (approval number B202505018; 25 February 2025), and all procedures conformed to the Declaration of Helsinki. Written consent was obtained before randomization. Participants were informed that participation was voluntary and that withdrawal would have no effect on their employment or working conditions. Data were handled confidentially and anonymously.

## 3. Results

### 3.1. Sample Characteristics

Of the 125 nurses screened for eligibility, 12 did not respond to online follow‐up invitations and five declined to participate. The remaining 108 nurses were randomly and equally assigned to the experimental and control groups, with 54 participants in each group. All participants completed the intervention and follow‐up assessments. The CONSORT flow diagram is presented in Figure 2.

**FIGURE 2:**
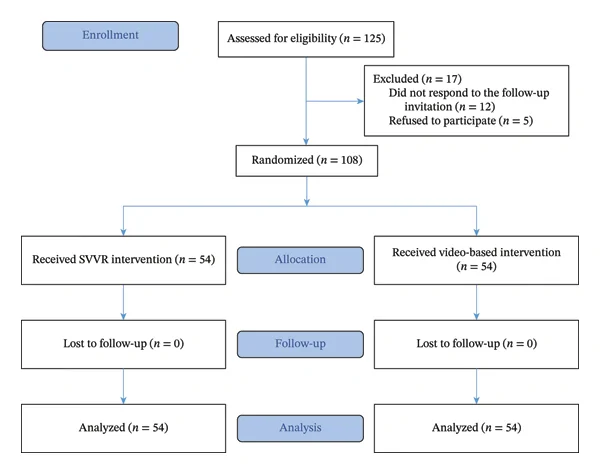
Flow of participant recruitment, randomization, group allocation, and inclusion in analysis. SVVR, spherical video‐based virtual reality.

The sample was predominantly female (83.3%), with a mean age of 31.57 years (standard deviation [*SD*] = 8.53). Most participants held a bachelor’s degree (74.1%) and worked in ICUs (*n* = 70, 64.8%). The mean length of clinical experience was 8.57 years (*SD* = 8.16; range = 0–37 years). Only 13 participants (12.0%) had previously received in‐service training on delirium care. Baseline comparisons showed no significant between‐group differences in demographic characteristics or baseline outcome variables, including delirium care knowledge, attitudes, and confidence (all *p* > 0.05), indicating adequate group balance (Table 2).

**TABLE 2:** Participant characteristics and baseline outcomes in the experimental and control groups.

Variable	Experimental (*n* = 54)	Control (*n* = 54)	*p*
*Demographic factors*			
Gender			0.439^a^
Male	11 (20.4)	7 (13.0)	
Female	43 (79.6)	47 (87.0)	
Age (years)	31.30 ± 8.09	31.85 ± 8.68	0.731^b^
20 − 30	37 (68.5)	35 (64.8)	0.339^a^
31 − 40	8 (14.8)	9 (16.7)	
41 − 50	9 (16.7)	7 (13)	
51 − 58	0 (0)	3 (5.6)	
*Education level*			0.501^a^
Diploma	7 (13.0)	5 (9.3)	
Bachelor	41 (75.9)	39 (72.2)	
Master	6 (11.1)	10 (18.5)	
Years of nursing practice	8.39 ± 7.67	8.75 ± 8.69	0.819^b^
Unit type			0.150^a^
ICU	37 (68.5)	33 (61.1)	
Medical–surgical ward	9 (16.7)	17 (31.5)	
Recovery room	8 (14.8)	4 (7.4)	
Prior delirium care training	6 (11.1)	7 (13)	0.767^a^
Outcome variables			
Knowledge	7.24 ± 1.20	7.58 ± 1.30	0.158^b^
Attitudes	41.28 ± 4.31	40.96 ± 5.18	0.732^b^
Confidence	5.57 ± 2.01	5.93 ± 1.65	0.321^b^

*Note:* Categorical variables are presented as frequency (%), and continuous variables are presented as mean ± standard deviation.

Abbreviation: ICU, intensive care unit.

^a^Chi‐square test (Fisher’s exact test applied where expected counts were < 5).

^b^Student’s *t* test.

### 3.2. Intervention Outcomes

Figure 3 presents changes in delirium care knowledge, attitudes, and confidence across the three time points.

**FIGURE 3:**
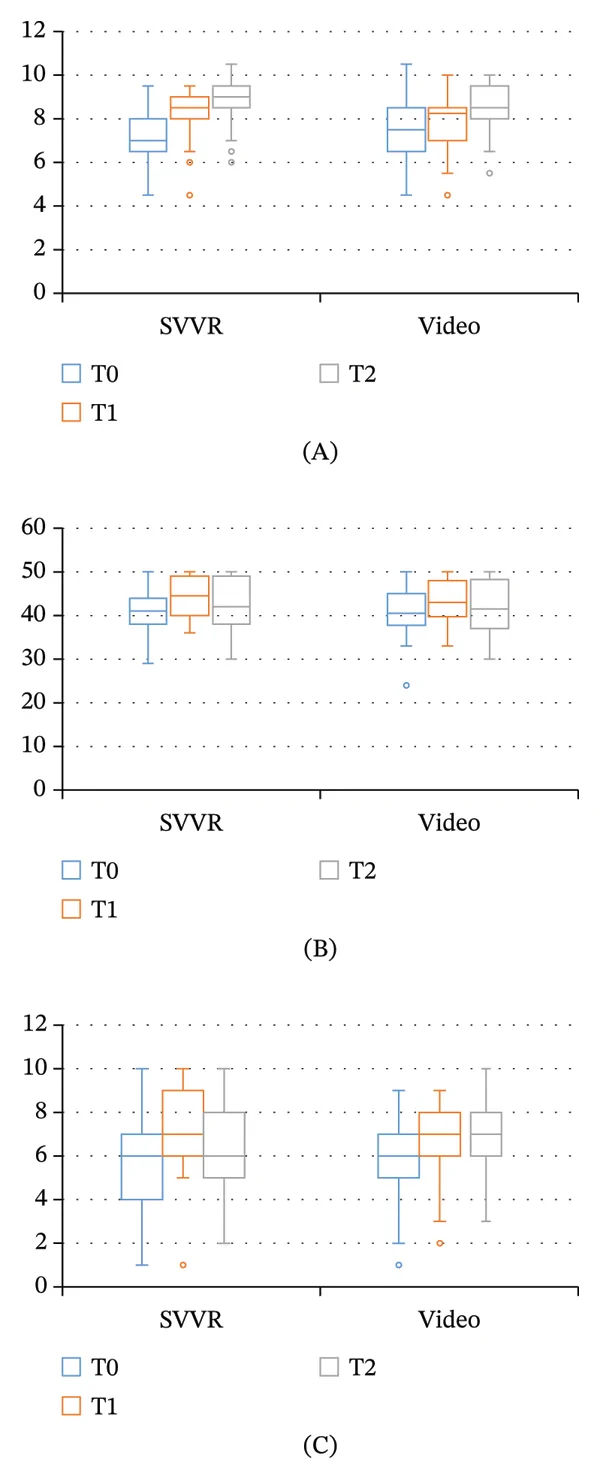
Within‐group changes in nurses’ delirium care competence across measurement points. Boxplots show within‐group changes in (A) knowledge, (B) attitudes, and (C) confidence toward delirium care at baseline (T0), immediately after the intervention (T1), and at 1‐month follow‐up (T2) in both the video and spherical video‐based virtual reality (SVVR) groups. The horizontal line within each box represents the median, the box indicates the interquartile range, and the whiskers represent the range of scores excluding outliers. Circles and asterisks indicate outlying values.

In the experimental group, knowledge scores increased from 7.24 at baseline to 8.29 immediately after the intervention and 8.79 at 1‐month follow‐up. Attitude scores increased from 41.28 to 44.30 immediately after the intervention, followed by a slight decline to 43.06 at follow‐up. Confidence scores increased from 5.57 to 7.30 immediately after the intervention and then slightly decreased to 6.39 at follow‐up.

In the control group, knowledge scores increased from 7.58 at baseline to 7.86 immediately after the intervention and 8.54 at follow‐up. Attitude scores increased from 40.96 to 43.39 immediately after the intervention and then declined to 41.98 at follow‐up. Confidence scores increased from 5.93 to 6.80 immediately after the intervention and remained higher than baseline at follow‐up.

For delirium care knowledge, Table 3 shows significant group‐by‐time interactions at both T1 (*B* = 0.77, 95% *CI* [0.32, 1.21], *p* = 0.001) and T2 (*B* = 0.58, 95% *CI* [0.09, 1.08], *p* = 0.022), indicating that increases from baseline were greater in the experimental group than in the control group. The between‐group effect was moderate immediately after the intervention (Cohen’s *d* = 0.54) and small to moderate at follow‐up (*d* = 0.32).

**TABLE 3:** Generalized estimating equation analysis of changes in delirium care knowledge, attitudes, and confidence by group and time.

Parameter	B	SE	95% CI	*χ* ^2^	*p*
Knowledge of delirium care					
Intercept	7.58	0.18	7.24 to 7.93	1865.82	< 0.001
Group^a^	−0.34	0.24	−0.81 to 0.13	2.06	0.15
Time					
T1 vs. T0^b^	0.28	0.16	−0.04 to 0.60	2.85	0.091
T2 vs. T0^b^	0.96	0.20	0.58 to 1.35	24.38	< 0.001
Group × Time					
SVVR × T1^c^	0.77	0.23	0.32 to 1.21	11.45	0.001
SVVR × T2^c^	0.58	0.25	0.09 to 1.08	5.26	0.022
Attitudes toward delirium assessment					
Intercept	40.96	0.70	39.59 to 42.33	3436.24	< 0.001
Group^a^	0.32	0.91	−1.47 to 2.10	0.12	0.729
Time					
T1 vs. T0^b^	2.43	0.52	1.41 to 3.45	21.69	< 0.001
T2 vs. T0^b^	1.02	0.62	−0.20 to 2.23	2.70	0.100
Group × Time					
SVVR × T1^c^	0.59	0.71	−0.81 to 1.99	0.69	0.406
SVVR × T2^d^	0.76	0.96	−1.12 to 2.64	0.63	0.428
Confidence in delirium management					
Intercept	5.93	0.22	5.49 to 6.36	712.58	< 0.001
Group^a^	−0.35	0.35	−1.04 to 0.33	1.01	0.315
Time					
T1 vs. T0^b^	0.87	0.17	0.55 to 1.20	27.58	< 0.001
T2 vs. T0^b^	0.72	0.20	0.32 to 1.12	12.58	< 0.001
Group × Time					
SVVR × T1^c^	0.85	0.25	0.36 to 1.35	11.32	0.001
SVVR × T2^d^	0.09	0.34	−0.58 to 0.77	0.07	0.787
Confidence in delirium management, adjusted model					
Intercept	5.51	0.36	4.81 to 6.21	235.44	< 0.001
Group^a^	−0.35	0.35	−1.03 to 0.33	1.03	0.310
Time					
T1 vs. T0^b^	0.84	0.27	0.30 to 1.38	9.36	0.002
T2 vs. T0^b^	0.15	0.38	−0.59 to 0.90	0.17	0.685
Group × Time					
SVVR × T1^c^	0.85	0.25	0.36 to 1.35	11.32	0.001
SVVR × T2^d^	0.09	0.34	−0.57 to 0.75	0.08	0.783
DCE	0.58	0.38	−0.17 to 1.32	2.32	0.128
Time × DCE					
Had DCE × T2^e^	0.79	0.38	0.03 to 1.54	4.20	0.040

*Note*: The effects were analyzed using a generalized estimating equation model. T0 indicates baseline; T1, immediately after the intervention; T2, 1‐month follow‐up; SVVR, spherical video‐based virtual reality; and DCE, delirium care experience during the period from T1 to T2. The control group and T0 were used as the reference categories. Data are presented as regression coefficient (*B*), standard errors (*SEs*), 95% confidence intervals (*CIs*), and Wald *χ* ^2^ *p* values. The symbol × indicates an interaction term.

^a^Baseline differences between two groups, with the video group as the reference category.

^b^Differences across time points, with T0 as the reference category.

^c^Group‐by‐time interaction at T1, with video group × T0 as the reference category.

^d^Group‐by‐time interaction at T2, with video group × T0 as the reference category.

^e^Time‐by‐DCE interaction at T2, with no DCE × T1 as the reference category.

Attitude showed a significant overall improvement at T1 (*B* = 2.43, 95% *CI* [1.41, 3.45], *p* < 0.001), whereas the group‐by‐time interactions at T1 and T2 were not statistically significant.

For confidence, the SVVR × T1 interaction was significant (*B* = 0.85, 95% *CI* [0.36, 1.35], *p* = 0.001), indicating greater immediate confidence gains in the experimental group. However, the SVVR × T2 interaction was not statistically significant (*B* = 0.09, 95% *CI* [−0.58, 0.77], *p* = 0.787).

### 3.3. Sensitivity Analysis

During the 1‐month follow‐up period, 78 participants (72.2%; 39 in each group) reported caring for patients with delirium, whereas 30 participants (27.8%) did not. After adjustment for postintervention delirium care experience, participants who had cared for patients with delirium reported higher confidence at follow‐up than those without such experience (*B* = 0.79, 95% *CI* [0.03, 1.54], *p* = 0.040). This finding suggests that real‐world clinical exposure may further reinforce nurses’ confidence in delirium management.

### 3.4. Tolerability of SVVR Training

Among the 54 participants in the experimental group, 18 (33.3%) reported no discomfort after the SVVR training. Among those who reported discomfort, total Virtual Reality Sickness Questionnaire scores ranged from 3.33 to 47.50, with a mean score of 15.85. The mean oculomotor and disorientation subscale scores were 10.78 and 5.07, respectively. No participant discontinued the intervention because of physical discomfort, and no serious adverse events were reported.

### 3.5. Qualitative Learning Experiences

All 54 participants in the experimental group participated in semistructured interviews. Thematic analysis identified three major themes and 12 subthemes describing nurses’ learning experiences with the SVVR delirium care training program. The qualitative findings were used to contextualize the observed improvements in delirium care competence. Themes, subthemes, and representative quotations are presented in Table 4.

**TABLE 4:** Themes and subthemes of participants’ learning experiences with spherical video‐based virtual reality training.

Themes	Subthemes	Supporting quotations	Participants
Advantages of spherical video‐based virtual reality training	Immersive experience	‘It’s an immersive approach that makes people feel they can more quickly transition and apply their learning to clinical practice.’	N1
		‘The virtual reality method makes you feel like you’re actually there; it’s quite realistic and feels just like being in a real hospital ward.’	N10
		‘You can see the surrounding environment more clearly, making it more immersive. In clinical situations, you can better express your immediate thoughts and consider what actions to take in response. It’s different from the detached feeling you get when watching videos—it feels more like an actual clinical learning experience.’	N29
	Enhanced concentration	‘I think it helps people become more immersed in the situation, allowing them to learn better and transform the experience into their own knowledge.’	N29
		‘You pay more attention to understand what is happening in the video; it’s more three‐dimensional. You also become more serious about it. Since you need to choose an answer right away and express your thoughts, you watch the video more attentively.’	N67
		‘This method forces you to pay serious attention to the virtual reality content.’	N98
	Deep impression	‘You need to see it in person to understand what delirium is and how nurses deal with it. I think this kind of learning method leaves a deeper impression and makes it easier to absorb.’	N6
		‘It is easier to remember the practice content and lesson materials, and it helps you to stay focused and absorb knowledge more effectively.’	N18
	Enhanced learning motivation	‘Seeing images and videos through the virtual reality method can better enhance the desire to learn.’	N13
		‘With more diverse options, nurses can choose their preferred learning methods, which will lead to more clinic nurses being willing to gain new knowledge.’	N23
		‘Virtual reality is uncommon, so people might think it is really cool and want to use this method for learning.’	N40

Knowledge gains about delirium care	Better understanding of assessment tools	‘Using virtual reality can help people quickly understand how to conduct assessments and help them remember which tools can be used to assess delirium.’	N2
		‘Using the assessment form through virtual reality helped me clarify things. Previously, when we were checking assessment tools, we would get somewhat confused. Now, I have a better understanding of delirium.’	N35
		‘I received some training on delirium assessment, and it served as a review for me on how to assess delirium.’	N71
	Familiarity with delirium management process	‘Through virtual reality technology, we can observe the entire process of patient delirium and see which immediate interventions we should provide. This helps us truly understand how we should handle such situations when we actually encounter them in clinical settings.’	N37
		‘I think this is quite good because it clearly explains what needs to be done—guiding us through the process step by step, suggesting what actions can be taken, and ultimately helping us gain a better understanding of the whole procedure.’	N71
		‘Through this virtual reality simulation approach, everyone can gain a clearer understanding of how to handle delirium situations.’	N87
	Opportunities for self‐reflection	‘When a patient experiences delirium, you can immediately see the patient’s current clinical signs. In that moment, you can start reflecting on what you did and did not do. In the final scene involving interprofessional collaboration, you can begin to reflect on yourself and think about what you might have missed or failed to communicate.’	N3
		‘It presents a realistic clinical simulation and allows you to pause the video, giving you time to reflect and learn simultaneously. It lets you stop and think about what you should do at that particular moment or how your actions should differ from the demonstrations shown in the video. People who watch videos typically just watch straight through, but this method forces you to pause and reflect.’	N31
		‘It’s about reflecting on your own work and considering whether, when a patient encounters these problems next time, you should address the problems rather than ignoring them.’	N76
	Understanding interprofessional collaboration	‘It helps you understand what other professionals such as nutritionists and respiratory therapists consider when it comes to delirium and helps you become more familiar with ways to collaborate with other teams.’	N3
		‘It made me realize that caring for a patient is not solely the responsibility of doctors and nurses. Things like electrolyte management, nutritional support, medication adjustments, and even respiratory exercises for intubated patients require the collaboration of various professionals.’	N6
		‘It helps you understand the interventions performed by each medical team and how they assess the patient.’	N11

Areas for improvement	Image resolution	‘The audio quality is acceptable, but the image quality is noticeably lacking.’	N1
		‘From certain angles, the image becomes somewhat blurry.’	N3
		‘Sometimes, it gets a bit blurry.’	N71
	Speech recognition	‘For speech recognition, it seems some parts could not be captured completely.’	N9
		‘During the recording process, it might not be able to fully present everything. Or, if we pause for too long while speaking, the recording gets interrupted.’	N20
		‘Some words of the live speech seemed to have not come through clearly.’	N28
	Equipment limitations	‘The equipment is quite user‐specific. For instance, since I have severe near‐sightedness, the limitations of the equipment may have had an impact.’	N29
		‘I have astigmatism, and it can be adjusted for near‐sightedness, but it would be good if it could be corrected for astigmatism too; the interface appeared quite blurry when I looked at it.’	N107
		‘Some more senior nurses might have presbyopia, so they may sometimes need to be further away to see the text descriptions clearly.’	N50
	Physical side effects	‘I felt a bit dizzy when wearing it just now, and I get dizzy if I wear it for too long.’	N44
		‘When I first put it on, there was a feeling of pressure in my eyes, as if the eye pressure was slightly higher. And occasionally, I feel a bit dizzy.’	N56
		‘I feel slightly dizzy when looking at things.’	N89

## 4. Discussion

Compared with the noninteractive video group, nurses who received the SVVR intervention showed significantly greater improvements in delirium care knowledge both immediately after training and at the 1‐month follow‐up. They also demonstrated a greater immediate improvement in confidence. Sensitivity analysis further indicated that nurses who provided delirium care during follow‐up had higher confidence, suggesting that clinical exposure may reinforce learning. The training was generally well tolerated, with only mild discomfort reported and no discontinuation due to adverse effects. Qualitative findings showed that the immersive format enhanced engagement, concentration, perceived realism, and clinical relevance.

The greater improvement in delirium care knowledge among nurses receiving SVVR training suggests that immersive and interactive learning may strengthen knowledge acquisition beyond conventional video‐based education. This finding is consistent with previous studies showing that SVVR training can improve learning performance, clinical reasoning, and knowledge retention in nursing and healthcare education [31, 32]. The sustained knowledge gains at 1‐month follow‐up may reflect the value of immersive clinical scenarios in helping nurses connect abstract delirium concepts with realistic patient assessment and management situations [33]. From the perspective of Bandura’s self‐efficacy theory, SVVR may provide vicarious learning and simulated mastery experiences that support continued learning and confidence in applying delirium care knowledge [19].

Both groups showed short‐term improvements in attitudes toward delirium care, but no significant between‐group differences were observed. This suggests that structured delirium education, regardless of delivery format, may improve nurses’ awareness of delirium assessment and management. However, attitude scores declined at 1‐month follow‐up, indicating that a single educational session may be insufficient to sustain attitudinal change [19]. Because attitudes may mediate the translation of knowledge into practice [34], nurse managers should consider repeated training, unit‐level reminders, and integration of delirium assessment into routine workflow.

Overall, the degree of confidence increased in both groups, with a greater immediate gain noted in the SVVR group. This finding suggests that immersive and interactive delivery may provide additional educational benefits beyond content exposure alone [20]. However, the between‐group difference was not sustained at 1‐month follow‐up. Sensitivity analysis revealed that nurses who cared for patients with delirium during the follow‐up period reported greater confidence than those without such experience, suggesting that subsequent real‐world clinical experience may reinforce confidence after formal training. According to Bandura’s self‐efficacy theory, confidence increases when individuals gain mastery through repeated practice and successful performance [19]. This indicates that SVVR and clinical exposure may have complementary functions. Specifically, SVVR may facilitate structured simulated mastery and preparation before clinical encounters, and direct patient care may provide authentic mastery experiences that reinforce confidence. This complementarity may be particularly essential in delirium care because fluctuating or hypoactive manifestations can be easily overlooked. Familiarity with key signs and assessment cues acquired through structured education may increase nurses’ attentiveness to clinical features suggestive of delirium and support more confident assessment when cases are subsequently encountered in practice. However, because delirium care exposure during this study’s follow‐up was naturally occurring rather than experimentally assigned, the present study cannot determine whether increased clinical exposure alone produces confidence gains comparable to those associated with SVVR training.

The qualitative findings help explain these quantitative results. Participants reported that SVVR created a realistic and engaging learning environment, enhanced concentration, and supported understanding of patient assessment, clinical decision‐making, and interdisciplinary collaboration. These experiences suggest that immersive training may help bridge the gap between classroom‐based education and complex clinical practice [35]. Participants also identified areas for improvement, including system functionality, image resolution, and interaction design. Future development should therefore focus on usability, technological quality, and integration with debriefing or guided reflection.

### 4.1. Implications for Nursing Management

From a nursing management perspective, SVVR may offer a feasible strategy for workforce development and continuing education. Delirium education can be challenging to deliver consistently through bedside teaching because delirium is unpredictable and fluctuating, which limits opportunities for direct observation and supervised practice. SVVR may address this challenge by providing standardized, repeatable, and realistic clinical scenarios without involving actual patients [20]. SVVR may serve as an initial preparation strategy, particularly for newly graduated nurses, to reinforce delirium assessment skills and support quality improvement initiatives related to early delirium recognition and patient safety. However, the implementation of SVVR requires consideration of equipment availability, initial development and maintenance costs, technical support, and staff familiarity with virtual reality technology. In contrast to SVVR‐based approaches, conventional video‐based education generally requires fewer technological resources and is accessible using commonly available devices such as computers or tablets, which potentially renders it easier to implement at scale. However, once developed, SVVR materials may offer flexibility in terms of scheduling and therefore may support self‐directed learning. Future studies should evaluate implementation outcomes, resource requirements, and cost‐effectiveness, as well as whether educational gains are sustained and translate into measurable changes in clinical practice.

### 4.2. Limitations

Several limitations should be considered when interpreting these findings. First, the single‐center sample, which consisted largely of ICU nurses, may restrict generalizability. Second, the 1‐month follow‐up period adopted in this study limits drawing of conclusions regarding the durability of knowledge gains and confidence, as well as whether these educational effects translate into sustained changes in clinical practice. Third, neither the participants nor the intervention team were blinded to group assignments, and the outcomes were primarily based on self‐report measures and knowledge tests rather than direct observations of clinical performance. In particular, the study did not directly assess behavioral change, delirium detection accuracy, or patient outcomes; therefore, whether the observed educational gains translated into improvements in clinical practice remains uncertain. Finally, the SVVR materials and equipment used require further refinement to enhance image quality, interaction design, and accessibility.

## 5. Conclusion

Our results indicate that SVVR training was associated with greater gains in delirium care knowledge and immediate confidence among nurses compared with noninteractive video‐based education. They also reveal that the knowledge gains achieved were sustained at 1‐month follow‐up and that the nurses’ confidence appeared to be reinforced by subsequent clinical exposure. These findings suggest that SVVR can be employed as a useful strategy in continuing education for delirium care. However, because this study did not assess clinical performance, delirium detection accuracy, or patient outcomes, further research with a longer follow‐up duration is required to determine whether these educational gains are sustained and translate into measurable changes in clinical practice and patient outcomes.

## Author Contributions

Yin‐Ling Hung and Yi‐Chao Zhou contributed to the study conception and design, definition of intellectual content, literature search, clinical and experimental studies, data acquisition and analysis, statistical analysis, and manuscript preparation and editing. Hsin‐Pei Feng and Chia‐Huei Lin contributed to the study conception and design, definition of intellectual content, literature search, and clinical studies. Wu‐Chien Chien contributed to the study conception and design, definition of intellectual content, literature search, data analysis, and statistical analysis. Wen‐Chii Tzeng contributed to the study conception and design, definition of intellectual content, literature search, clinical and experimental studies, data acquisition, and manuscript preparation and editing, and served as the guarantor of the work.

## Funding

This work was supported by the National Science and Technology Council, Taiwan (grant number: NSTC‐113‐2314‐B‐016‐019‐MY3) and Medical Affairs Bureau Ministry of National Defense, Taiwan (grant number: MND‐MAB‐D‐115159).

## Disclosure

All authors reviewed the manuscript. The spherical video‐based virtual reality equipment used in this study was provided by National Defense Medical University. The institution had no role in the study design, data collection, data analysis, interpretation of findings, decision to submit the article for publication, or manuscript preparation.

Organization: Sigma’s 36th International Nursing Research Congress.

Place: Seattle, Washington, USA.

Date: July 2025.

## Ethics Statement

The study was conducted in accordance with the Declaration of Helsinki and approved by the Institutional Review Board of Tri‐Service General Hospital, National Defense Medical University (Approval No.: B202505018; approval date: 25 February 2025).

## Conflicts of Interest

The authors declare no conflicts of interest.

## Data Availability

The data that support the findings of this study are available from the corresponding author, Wen‐Chii Tzeng, upon reasonable request.
